# Löfgren Syndrome: A Mosaic of Sarcoidosis Phenotypes

**DOI:** 10.7759/cureus.52317

**Published:** 2024-01-15

**Authors:** Francisca Martins, Miguel Martins, Rui Malheiro

**Affiliations:** 1 Internal Medicine, Centro Hospitalar Universitário de Lisboa Central, Lisbon, PRT

**Keywords:** löfgren syndrome, sarcoid arthritis, subcutaneous sarcoidosis, panniculitis, acute sarcoidosis

## Abstract

Sarcoidosis is an autoimmune multisystemic granulomatous disease with an unknown etiology. Löfgren syndrome (LS), an infrequent initial presentation of acute sarcoidosis, is characterized by the classic triad of acute arthritis, erythema nodosum (EN), and bilateral hilar lymphadenopathy (BHL). The presence of this triad offers high diagnostic specificity for sarcoidosis, eliminating the need for a confirmatory biopsy. Typically, LS follows a predictable, self-limiting clinical course. However, atypical presentations require early suspicion and closer monitoring.

This case report highlights an unusual clinical manifestation of LS, marked by an incomplete presentation with acute panniculitis and joint lesions in the absence of EN. Acute sarcoidosis should be considered among the differential diagnoses when these clinical manifestations are present, and chest radiography should be performed to rule out BHL. In atypical cases, the disease course becomes less predictable, as exemplified in our case, where recurrence of the disease may occur, necessitating consistent monitoring.

## Introduction

Sarcoidosis, marked by noncaseating granuloma formation, is a complex multisystem disorder with the potential to affect numerous organs. Consequently, this disease can be presented with a range of symptoms, from none to severe organ problems, corresponding to different clinical phenotypes [1-6]. Sarcoidosis is a worldwide condition with varying incidence rates across different regions ranging from 1 to 15 cases per 100,000 people, with the highest rates observed in afro-descendants and Northern European countries, 11-15 cases per 100,000 people [1,7]. Variations in sarcoidosis incidence and prevalence rates are often attributed to disparities in genetics, environmental exposures, or differences in detection and diagnostic methods [1,5,8,9]. In many parts of the world, the true burden of sarcoidosis remains unclear due to the existence of diseases that mimic its symptoms (e.g., tuberculosis), inadequate access to diagnostic technology and knowledge, and limited case recording [2,8,9]. The diagnosis can be overlooked when clinicians are not familiar with its various presenting characteristics and the appropriate diagnostic evaluations involving imaging studies and tissue biopsies [8,10].

Sarcoidosis may be acute or chronic, with acute forms often displaying a positive prognosis, frequently achieving complete remission within the initial two years [8]. Certain clinical presentations exhibit such distinct symptoms that they have been recognized as syndromes. Löfgren syndrome (LS), consistently considered the most well-established phenotype of sarcoidosis, is characterized by the coexistence of bilateral hilar adenopathy on chest radiography, bilateral ankle arthritis (typically in men), and/or erythema nodosum (EN) (typically in women) [4]. LS has the highest reported incidence in individuals of white ethnicity and is rarely diagnosed in black or Asian individuals. In Sweden, the syndrome comprises approximately 30% of all sarcoidosis cases [4,7]. The majority of patients with LS experience spontaneous resolution within three to six months [5].

We present a case of bilateral diffuse panniculitis in a 36-year-old woman with systemic involvement, whose presentation and clinical course demonstrated to be a unique mosaic of sarcoidosis phenotypes. We reviewed the state of the art regarding clinical presentation, differential diagnosis, and natural history, highlighting LS. Given the idiosyncratic characteristics of this clinical scenario, we focused on cutaneous, subcutaneous, and musculoskeletal manifestations. We also discuss the timing of starting treatment and the expected evolution of the acute form of sarcoidosis.

## Case presentation

A 36-year-old Caucasian non-smoker female patient was referred to our internal medicine department for the investigation of pain and inflammatory signs in her bilateral tibiotarsal joints over the seven days that preceded her referral. The patient had initially noted painful swelling, redness, and stiffness in both her ankles early in March. Subsequently, she reported a progressive worsening of these conditions over the 48 hours preceding her referral, which had evolved and impaired her ability to walk, compelling her to use a wheelchair for movement. The pain reportedly became worse during times of rest, especially at night, and the morning stiffness lasted for more than two hours. Notably, the patient did not report the occurrence of fever, night sweats, weight loss, or other accompanying symptoms. She did not have any history of trauma, recent infections, travel, or risk behavior for sexually transmitted diseases. She worked as a healthcare professional, with no medical history, no record of drug addiction or medication use, and no known allergies prior to her referral.

**Figure 1:**
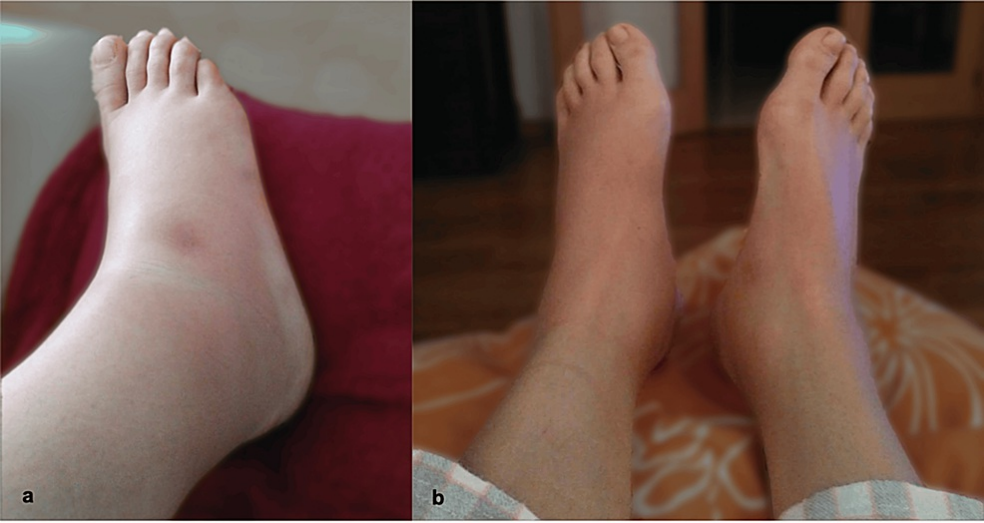
Photograph sent by our patient a day before her hospital admission, documenting the bilateral ankle panniculitis, a skin-colored subcutaneous swelling in lateral view of the right foot (a) and both feet (b).

The patient’s physical examination revealed exuberant inflammatory signs in both her feet, her tibiotarsal joints, and the lower thirds of her legs. Specifically, there was a fat pad anterior to the lateral malleoli, soft tissue non-pitting edema, and the absence of a Stemmer sign (Figure 1). As mentioned before, the patient encountered pain at rest and both passive and active movements were restricted in the affected joints. However, the examination of the other joints revealed no abnormality. Upon further examination, no palpable lymphadenopathies or organomegaly were detected in the accessible areas, and the patient’s cardiovascular, respiratory, and neurological systems appeared unremarkable.

The patient’s admission blood samples showed an elevated erythrocyte sedimentation rate (71 mm/h), the presence of C-reactive protein (159 mg/L), normal serum angiotensin-converting enzyme, and protein electrophoresis. The autoimmune disease-related studies revealed a negative report regarding HLA-B27, antinuclear antibodies, anti-citrullinated protein antibodies, and rheumatic factors (Table 1). The screenings for tuberculosis and other infectious diseases also showed negative reports.

**Table 1:** Laboratory findings on admission, six months after admission, and recurrence after 12 months. anti-CCP Ab, anti-cyclic citrullinated protein antibody; HBsAg, hepatitis B surface antigen; anti-HCV total, hepatitis C virus antibody; Ac Anti-HIV 1 + 2: human immunodeficiency virus types 1 and 2 antibodies; RPR, rapid plasma reagin; VDRL, venereal disease research laboratory

	Reference values	Admission	Six months after admission	Recurrence after 12 months
﻿White blood cell (/L)	4.5 - 11.0× 10^9^	9.22 × 10^9^	7.10 × 10^9^	7.57 × 10^9^
﻿Neutrophils (%)	40 - 75	80.3	53.4	85.4
﻿Hemoglobin (g/dL)	12.0 - 15.0 × 10	12.6 × 10	13.8 × 10	14.5 × 10
﻿Platelet (/L)	150 - 450 × 10^9^	298 × 10^9^	257 × 10^9^	290 × 10^9^
﻿Blood urea nitrogen (mg/dL)	15.0 - 40.0	14	39	34
﻿Creatinine (mg/dL)	0.57 - 1.11	0.64	0.74	0.77
﻿Protein (g/L)	60 - 83	67.8	74.9	-
﻿Albumin (%)	55.8 - 66.1	48.8	60.9	
﻿Aspartate aminotransferase (IU/L)	5.0 - 34.0	14	27	17
﻿Alanine aminotransferase (IU/L)	0.0 - 55.0	18	22	24
﻿Alkaline phosphatase (IU/L)	40 - 150	62	67	74
﻿Angiotensin-converting enzyme (U/L)	16 - 85	25	20	63
﻿Erythrocyte sedimentation rate (mm/hr)	<16	71	6	20
﻿C-reactive protein (mg/L)	<5.0	175.9	1.3	80.8
﻿Rheumatoid factor (U/mL)	<15	<12.5	-	-
﻿Anti-nuclear antibodies	NA	Negative	-	-
﻿Anti-CCP Ab (UQ)	<20	<4.6	-	-
HBsAg	NA	Negative	-	-
Anti-HCV total	NA	Negative	-	-
Ac anti-HIV 1 + 2	NA	Negative	-	-
RPR (VDRL)	NA	Negative	-	-

**Figure 2:**
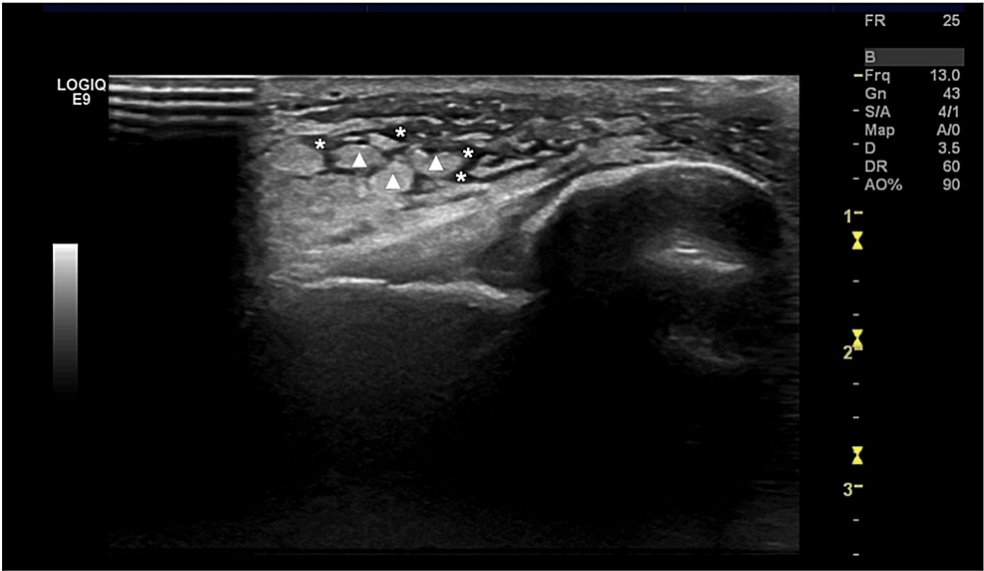
Grayscale ultrasound showing hypoechoic subcutaneous tissue (asterisks), decreased echogenicity of the dermis, and increased size and echogenicity of the fatty lobules (triangles) of the subcutaneous tissue.

Moreover, the ultrasound of the patient’s tibiotarsal joints showed the following: diffuse subcutaneous edema of the feet and tibiotarsal joints, with more significant edema on the left foot; moderate tenosynovitis of the peroneal and posterior tibial tendons, and the absence of joint effusion or appreciable synovial thickening of the tibiotarsal or intertarsal joints. No micro- or macro-vascular changes were observed (Figure 2). 

**Figure 3:**
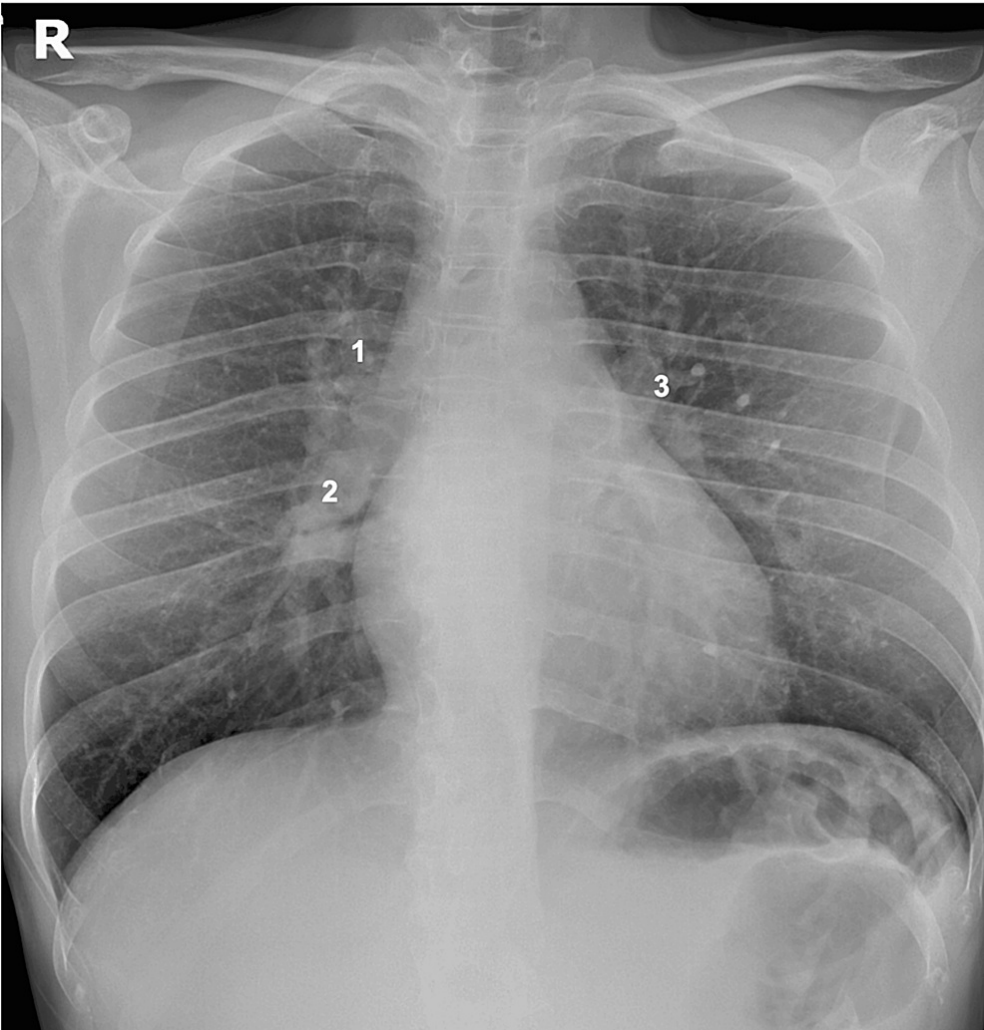
A PA chest x-ray revealing the classic pattern of sarcoidosis: 123 sign or Garland's triad. The characteristics of this sign include mediastinal enlargement, usually right paratracheal (1) and bilateral hilar nodes, on the right (2) and left (3), due to lymphadenopathy. PA, posteroanterior

**Figure 4:**
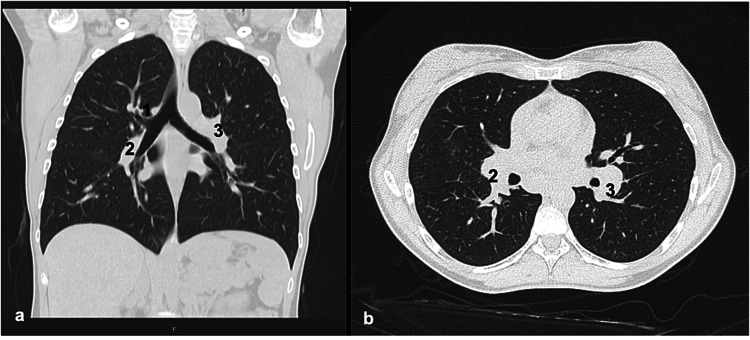
Contrast thoracic CT, in coronal (a) and transverse (b) plane, revealing a classic 123 sign with bilateral hilar (2 and 3) and paratracheal (1) lymphadenopathy, with no other abnormalities. CT, computed tomography

Notably, the patient’s chest X-ray film revealed bilateral hilar lymphadenopathy (BHL) (Figure 3). Furthermore, a contrast thoracic abdominal computed tomography (CT) revealed a classic "123 sign" with bilateral hilar and paratracheal lymphadenopathy but no other observable abnormality (Figure 4). 

**Figure 5:**
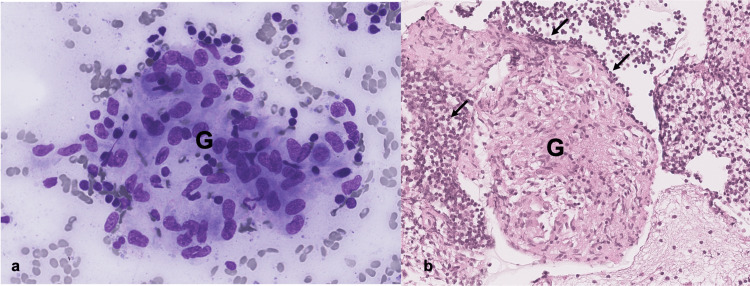
(a) Microscopic view of a granuloma (G), highlighting immune cells such as macrophages and lymphocytes in distinctive violet hues following Giemsa staining; (b) microscopic view of a granuloma structure within a cell block sample, visualized after hematoxylin and eosin staining, showcasing the distinctive cellular arrangement and morphology of a non-caseating granuloma (G), on a background of lymphocytes (arrows). Biopsy slides’ images were provided to the Pathology Department team at the Portuguese Oncology Institute, Lisbon.

To confirm the diagnosis and rule out other potential causes of lymph node enlargement, bronchoalveolar lavage (BAL) and endobronchial ultrasound-guided transbronchial needle aspiration (EBUS-TBNA) were conducted on the hilar and mediastinal lymph nodes. The obtained material underwent microbiological examination, including special staining for fungi and acid-fast bacilli, as well as cultures for tuberculosis and fungi, all of which returned negative results. A polymerase chain reaction (PCR) test for acid-fast bacilli was also performed and yielded a negative result. CD4:CD8 T lymphocyte ratio was not performed. Pathological analysis revealed well-formed non-necrotizing granulomas, characterized by epithelioid histiocytes accompanied by lymphocytes (Figure 5).

After the diagnosis of sarcoidosis was established, the patient started prednisolone at a dosage of 0.5 mg/kg/day. Remarkably, there was a noticeable improvement in pain within two days, and signs of inflammation in the soft tissues improved over three weeks, resulting in the restoration of the patient's ability to walk.

After one month of steroid treatment, the patient initiated the tapering process and eventually discontinued the treatment after six months. During the period of corticosteroid therapy, no infections were observed; however, the patient reported an 8% increase in weight gain. Upon the six-month and nine-month follow-up evaluation, the patient exhibited full recovery, and the objective examination revealed no positive findings. An analytical assessment demonstrated a regression of inflammatory markers (Table 1) and a subsequent chest X-ray showed no signs of lymphadenopathy.

The patient maintained remission for one year, free of symptoms or limitations, and with normal results in additional tests. However, after this period, the patient reported a recurrence of fatigue following minor exertion, along with panniculitis and morning arthralgia in the ankle. The stiffness persisted for more than two hours, significantly impacting daily functioning and hindering professional activities. A chest CT revealed bilateral lymphadenopathy, and laboratory data indicated increased inflammatory markers (Table 1).

The patient expressed significant concerns about the professional implications of her limitations and the weight gain she had experienced in the past due to corticosteroid therapy. After carefully weighing the associated risks and benefits, a collaborative decision was made with the patient to initiate low-dose methotrexate at 10 mg once weekly. Remarkably, the patient's complaints were resolved within the next four weeks. Currently, the patient is under our ongoing care, and her symptoms are effectively controlled with continuous therapy.

## Discussion

The above clinical case describes a rare acute manifestation of sarcoidosis. The diagnostic criteria of sarcoidosis include a combination of clinical and radiological presentation, the presence of non-caseating granulomas, and the complete exclusion of alternative diseases [1-3]. The respective weight of each criterion varies depending on the presentation and evolution of sarcoidosis [5]. 

In 1952, a triad of sarcoidosis-related acute symptoms was described: BHL, EN, and/or bilateral ankle arthritis or periarticular inflammation (PAI), which became known as LS [8,10]. Unlike the chronic groups, which preferentially affect individuals of African descent, LS predominantly affects individuals of Caucasian European descent, with a significant presence in Sweden and the Netherlands [7]. Thus, numerous research groups have concentrated on delineating the phenotypic patterns to link them with distinct genetic backgrounds and underlying biological pathways, with the aim of predicting clinical progression and treatment response [11-13].

﻿EN is a reactive nodular inflammatory panniculitis that develops in up to 25% of patients with sarcoidosis and serves as the primary cutaneous manifestation of LS. It is considered as a non-specific cutaneous manifestation of sarcoidosis, as histological examination does not reveal granulomas [14,15]. EN is characterized by erythematous, violet, or brown subcutaneous nodules that are tender and warm to the touch. These nodules are typically found in the pre-tibial areas of the lower limbs and are frequently associated with symptoms such as arthralgia, periarteritis, lower limb edema, and fever [14,15]. Importantly, they have been associated with a favorable prognosis [1-10,14,15]. 

During the initial evaluation, our medical team initially assumed that the skin manifestations in our patient could signify EN. The absence of palpable nodules was attributed to the presence of significant periarticular edema. However, a subsequent ultrasound examination did not confirm this suspicion. The prominent PAI observed in our patient was described as non-septal panniculitis, representing a rare and non-specific subcutaneous manifestation of sarcoidosis [14,15]. Indeed, contrary to EN, the lesions in subcutaneous sarcoidosis are not tender, have a flesh-colored appearance, may persist for extended periods, and are strongly associated with mild systemic involvement [14,15].

Hence, following a clinical suspicion, a chest X-ray was performed, which confirmed radiographic stage I according to the Scadding chest X-ray staging system, characterized by bilateral mediastinal and hilar adenopathy without pulmonary infiltrate, as observed in the majority of described patients in the medical literature (79%-82.5%) [13].

In fact, the presence of isolated PAI in conjunction with BHL (without EN) remains documented by Caplan et al. and also in the more recent studies; the proportion of the relevant patients goes up to 6.4% in some of the series [10-13]. In contrast to the typical presentation of LS, this variant is observed more frequently in men in the 25-40 age range and tends to manifest during the spring season, which could indicate a set of environmental associations related to the etiology of this variant [5,7,10,11,13].

Joint involvement in sarcoidosis remains recorded in 2%-38% of cases [16,17]. Overall, joint involvement in the context of LS is described as migratory polyarthritis, often symmetrical and predominantly affecting the large joints, with the ankle joint being involved in over 90% of the relevant cases. Joint pain is often the main complaint of patients with LS, which leads them to seek medical attention. Most patients initially exhibit stiffness and pain, often describing the same as a dull ache [18,19]. In this regard, the most notable clinical observation is the predominant location of the inflammation in the tissues surrounding the joints (periarteritis) [16,18,19]. This manifestation is characterized by moderate to severe soft tissue swelling and tenderness in 70% of patients with ankle involvement [16,20]. In many cases, the associated redness and warmth of the skin resembles cellulitis. Moreover, tenderness often persists even after the swelling subsides. In contrast to this PAI, intraarticular involvement is relatively minor. In the cases described in the medical literature, the pain remains minimal or non-existent during active or passive movement of the joint and does not compromise walking, even when the ankles are acutely inflamed [18,19]. Ultrasonography typically indicates the swelling of the soft tissues surrounding the joints and tenosynovitis, with joint effusion or synovitis being observed less frequently [17,18]. In Caplan’s series, only one of the 19 patients reports severe pain during joint movements, similar to the claims of our patient [10].

In the case presented in this report, the clinical and ultrasound findings of our patient appeared to align with the abovementioned rare subgroup described by Caplan et al. [10]. However, to confirm the diagnosis of sarcoidosis, it was still necessary to identify non-caseating granulomas and rule out other pathologies. Indeed, in cases where the triad that indicates LS is present, the diagnosis is usually straightforward, with high specificity (93%); in this regard, histological confirmation is not mandatory [20]. However, in some instances, the diagnosis of LS can be challenging due to the presence of variant forms of the syndrome or an incomplete initial presentation, both of which make it difficult to exclude other potential pathologies and establish the correct diagnosis. This was the exact situation in our case [8]. Notably, tuberculosis can involve a particularly challenging differential diagnosis, not only in terms of pulmonary presentations but even in cases involving arthritis presentations, as exemplified by the need to differentiate it from Poncet’s Disease [4]. Thus, given the prevalence of tuberculosis in Portugal and the related risk of potential occupational exposure for our patient, it was crucial to rule out tuberculosis at the outset.

When performing a biopsy on the skin or joints, the typical findings often involve mild, non-specific inflammation of the synovium, characterized by the presence of mononuclear cells surrounding blood vessels within the synovial tissue or panniculitis; the presence of non-caseating granulomas is rare [9]. In our case, due to the limited precision of the skin and joint biopsies, we decided to proceed with a transbronchial biopsy. Here, one must note that EBUS-TBNA is currently the preferred diagnostic procedure regarding sarcoidosis in many medical centers due to its high sensitivity and low complication rate; in fact, meta-analyses have indicated a diagnostic yield for sarcoidosis ranging from 54% to 93%, with an overall sensitivity of 79% [2,5,6]. As per this procedure, the biopsy specimen must undergo a thorough evaluation to exclude other potential causes of granulomatous inflammation, especially fungal and mycobacterial infections, as well as foreign body reactions [4,8]. This evaluation includes specific staining and culture tests for mycobacteria and fungi. Moreover, BAL typically reveals a moderate lymphocytosis (20%-50%) in 80% of sarcoidosis cases and a CD4:CD8 T lymphocyte ratio greater than 3.5 in 50% of the cases, further supporting the diagnosis of sarcoidosis [4]. In our case, the biopsies in our patient confirmed the presence of non-caseating granulomas and ruled out other conditions.

Regarding sarcoidosis treatment, the best-defined guidelines primarily address pulmonary manifestations, as they are more frequent and have a significant impact on prognosis [1,2]. Managing the extrapulmonary manifestations of sarcoidosis is a complex issue, mainly due to the challenge of predicting disease progression, which can resolve spontaneously even in advanced cases [8,9]. The therapeutic options include disease-modifying medications such as steroids, classical immunosuppressants (e.g., methotrexate, azathioprine), and biologics (e.g., infliximab) [5,6]. Furthermore, the initiation of treatment should consider the disease’s severity or its potential to worsen, in addition to assessing how the symptoms affect a given patient’s quality of life [7,9].

Prednisolone, a glucocorticoid, remains the primary substance that is used in the treatment of sarcoidosis. One proposed regimen includes the administration of prednisolone at a dose of 0.5-0.75 mg/kg of one’s body weight on a daily basis for four weeks, followed by a tapering regimen that involves decreasing the dosage by 10 mg after every four-week period based on the disease response [4]. In many cases, treatment can be discontinued after six to 12 months, provided that the patients become asymptomatic and their lung function improves. Notably, regular monitoring is crucial, and experts recommend the continuance of vigilant post-treatment monitoring for at least three years after discontinuation [4].

Most patients with LS achieve remission; however, chronic forms of LS do occur, remaining active two years after diagnosis; more rarely (3%-6%), recurrences occur after remission [20]. In our patient’s case, after months of remission, a subacute condition developed along with extreme fatigue and arthralgias [8]. This situation could be compared to those described in the available medical literature, where one-third of patients reportedly exhibit constitutional symptoms, with fatigue being present in up to 90% of cases and significantly affecting their quality of life [1].

On the other hand, chronic arthritis is extremely rare, as inflammatory arthritis usually resolves within six weeks in most patients and within two years in almost all patients. A chronic course lasting for more than two years is observed in 8%-22.6% of patients with LS and is associated with advanced age, stage II sarcoidosis diagnosis, and the need for treatment [9,16]. Notably, chronic forms of sarcoid arthritis involve a less symmetrical distribution of joint and skin involvement compared to that of LS and are usually associated with either pulmonary or extrapulmonary parenchymal sarcoidosis [16].

Given the rarity of these forms, treatment decisions must be made on a case-by-case basis. In the case of our patient, as her symptoms had a substantial impact on functional capacity and the literature indicates favorable outcomes in managing cutaneous and joint manifestations, we opted to initiate weekly methotrexate therapy [2,5].

One must remember that sarcoidosis is associated with an increased risk of several comorbidities, including infections, heart failure, stroke, autoimmune diseases, and various types of cancer [20]. Since there are limited studies examining the contribution of glucocorticoids and other immunosuppressive agents as potential factors in the development of these comorbidities, it is challenging to distinguish the influence of the disease itself from the impact of treatment [4].

Still, the prognosis of sarcoidosis is generally favorable, with fewer than 10% of patients succumbing to the disease (mainly due to advanced lung involvement). Several factors, such as age at the time of diagnosis, disease presentation, pulmonary fibrosis, cardiac and neurological complications, and pulmonary hypertension, influence the prognosis of sarcoidosis [2,4,7]. Thus, careful management and monitoring are essential to improve the concomitant patient outcomes.

## Conclusions

This case report aims to underscore the uncommon presentation of LS, characterized by the absence of EN but featuring diffuse panniculitis. It highlights the necessity of recognizing the diverse manifestations of sarcoidosis. Particularly noteworthy is the clinical recurrence experienced by our patient, a deviation from the typical cases documented in the medical literature. In light of this, we emphasize the critical importance of ongoing disease monitoring and the implementation of personalized treatment strategies for sarcoidosis.
